# Too severe to save? Association between the timing of decompressive craniectomy and mortality in pediatric traumatic brain injury using the German hospital database

**DOI:** 10.1186/s13054-025-05488-3

**Published:** 2025-07-01

**Authors:** Rayan Hojeij, Pia Brensing, Bernd Kowall, Andreas Stang, Michael Nonnemacher, Ursula Felderhoff-Müser, Philipp Dammann, Marcel Dudda, Christian Dohna-Schwake, Nora Bruns

**Affiliations:** 1 Department of Pediatrics I, Neonatology, Pediatric Intensive Care Medicine, Pediatric Neurology, and Pediatric Infectious Diseases, University Hospital Essen, University of Duisburg-Essen, Essen, Germany; 2 Centre for Translational Neuro- and Behavioural Sciences, University Hospital Essen, C-TNBS, University of Duisburg-Essen, Essen, Germany; 3 Institute for Medical Informatics, Biometry and Epidemiology, University Hospital Essen, University of Duisburg-Essen, Essen, Germany; 4 Department of Neurosurgery and Spine Surgery, University Hospital Essen, University of Duisburg-Essen, Essen, Germany; 5 Department of Orthopedics and Trauma Surgery, BG Klinikum Duisburg, University of Duisburg-Essen, Duisburg, Germany; 6 Department of Trauma, Hand, and Reconstructive Surgery, University Hospital Essen, University of Duisburg-Essen, Essen, Germany

## Dilemma of timing in pediatric TBI

Decompressive craniectomy (DC) is a critical intervention for controlling intracranial pressure (ICP) in children suffering from severe traumatic brain injury (sTBI). However, the optimal timing for this procedure remains unclear, with a lack of clear clinical guidelines for both adult and pediatric populations.

Some studies suggest that earlier intervention in pediatric sTBI, based on varying time-to-surgery thresholds, may reduce ICP and improve outcomes, while others emphasize the potential risks of early intervention [1]. The timing of DC is particularly controversial in pediatric patients, where rapid deterioration and a smaller intracranial pressure reserve may complicate decision-making. Previous trials such as DECRA and RESCUEicp have provided important insights on the effects of DC in adults, showing that while DC can effectively lower ICP, it does not necessarily improve functional outcomes and may even be associated with higher rates of severe disability [2–4]. The literature lacks a universal definition for “early” or “late” DC, with cut-off times ranging from 2 to 24 h after admission or accident.

## Analysis from German hospital database (GHD)

The aim of this study was to investigate the association between timing of DC and in-hospital mortality in children with sTBI in Germany. We analyzed cases < 18 years of age from the German hospital database from 2016 to 2022 with sTBI who underwent DC. Selected cases had TBI as primary discharge diagnosis (ICD-10 code: S06) identified via codes of the International-Classification of Disease, 10 th edition, German modification (ICD-10-GM). Cases, procedures and time intervals were identified via ICD codes, procedure codes, and corresponding timestamps.

We defined sTBI as an Abbreviated Injury Scale (AIS) of the head ≥ 3. Injury severity was further assessed using survival risk ratios (SRR), and for each case, the lowest SRR was used to predict the severity of the injury for each case, (single ICISS: International Classification of Injury Severity Score), as previously described [5]. The outcome of interest was in-hospital mortality. A data driven approach was used to define time categories for the time from admission to DC of 0 to < 2 h, 2 to < 3 h, and ≥ 3 h.

### Main results

**Fig. 1:**
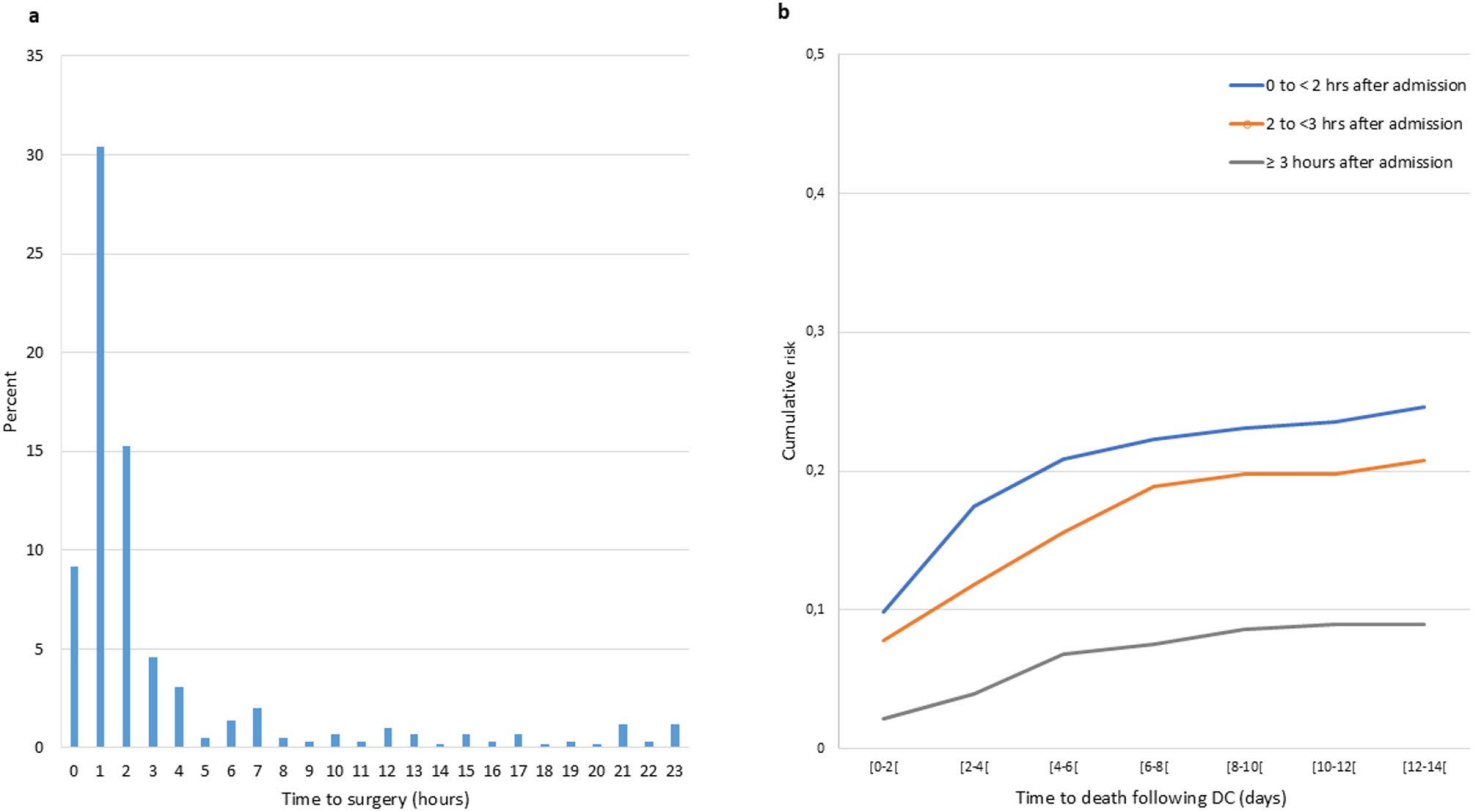
**a**. Distribution of time within the initial 24 h from admission to decompressive craniectomy surgery. *Hours are shown as complete hours. This graph represents 75% of the patients who underwent DC surgery in the first 24 h of admission. **b**. Cumulative case fatality following time to DC in hours by time to death within 14 days of hospitalization after the DC surgery. *Days and hours are shown as complete days/hours

9,495 severe TBI cases were identified with AIS head ≥ 3 with a median age of 12 years (IQR 4–16). Of these, 598 (6.3%) cases underwent DC during their hospital stay, 236 (40%) within the first hour and 443 (75.2%) within the first 24 h of hospitalization. More that the half of the DCs (54.8%) were performed within two hours of hospital admission, with numbers declining over the first 24 h of admission (Fig. 1). 164 (27%) of DC cases died in hospital with a median time from admission to death of 2 days. These findings suggest that a substantial proportion of DCs were performed during the initial shock room management and reflected urgent clinical decisions.

## Early DC mortality and injury severity

In-hospital mortality was highest among patients who underwent DC within the first hour of admission showing a mortality of 38.6%, followed by 34.4% in the 2- <3 h group and decreased to 15.8% in those who had surgery more than three hours after admission. The median single ICISS was lowest for the 0- <2 h group, at 0.65 (10 th and 90 th percentiles: 0.07–0.78), compared to 0.70 (0.33–0.87) for the 2- <3 h group and 0.69 (0.37–0.92) for the group undergoing surgery more than 3 h after admission. The cumulative case fatality within the first 14 days was higher for those who received earlier DC within the first hour of admission, compared to those who underwent DC after a delay of 2 to 3 h or more (Fig. 1).

The occurrence of early death also reflects the rapid clinical deterioration even after the surgical intervention, suggesting that these patients suffered from non-survivable injuries. Moreover, the fact that more than half of the procedures were performed during the initial shock room stay further supports the hypothesis of a severely compromised physiological status upon arrival. Finally, the most severe injury score were observed in the 0- <2 h group, suggesting that the severity of the initial injury and the associated complications could not be mitigated by the benefits of very early DC.

## Timing considerations for DC in pediatric TBI

These findings underscore the complexity of timing in pediatric trauma care, where the need for early intervention mirrors the severity of the condition. Performing the intervention does not guarantee survival and may be achieved at the cost of poor functional outcomes. Pediatric trauma teams must weigh the risks and benefits of early intervention, considering the specific characteristics of each case. To overcome this, further research is needed to determine which patients benefit from early DC and provide guidance for multidisciplinary decision-making in sTBI. A shift toward individualized, risk-based approaches could help to refine decision-making, and ultimately improve outcomes in this vulnerable population.

## Data Availability

The data analyzed in this study is subject to the following licenses/restrictions: the original dataset can be accessed after inquiry to the Federal Bureau of Statistics of Germany. Requests to access these datasets should be directed to https://www.forschungsdatenzentrum.de/de. Data sources: RDC of the Federal Statistical Office and the Statistical Offices of the Federal States, dataset DOIs :2016: 10.21242/23141.2016.00.00.1.1.0, 2017: 10.21242/23141.2017.00.00.1.1.0, 2018: 10.21242/23141.2018.00.00.1.1.0, 2019: 10.21242/23141.2019.00.00.1.1.1, 2020: 10.21242/23141.2020.00.00.1.1.0, 2021: 10.21242/23141.2021.00.00.1.1.0, 2022: 10.21242/23141.2022.00.00.1.1.0.

## Abbreviations

AIS: Abbreviated Injury Scale
DC: Decompressive craniectomy
GHD: German Hospital Database
ICD-10-GM: International-Classification of Disease, 10^th^ edition, German modification
ICISS: International Classification of Injury Severity Score
ICP: Intracranial pressure
SRR: Survival risk ratios
sTBI: Severe traumatic Brain Injury
